# Meta-Analysis of Effect Sizes Reported at Multiple Time Points Using General Linear Mixed Model

**DOI:** 10.1371/journal.pone.0164898

**Published:** 2016-10-31

**Authors:** Alfred Musekiwa, Samuel O. M. Manda, Henry G. Mwambi, Ding-Geng Chen

**Affiliations:** 1 School of Mathematics, Statistics, and Computer Science, University of KwaZulu-Natal, Pietermaritzburg, South Africa; 2 Biostatistics Unit, South African Medical Research Council, Pretoria, South Africa; 3 School of Social Work, University of North Carolina at Chapel Hill, Chapel Hill, NC, United States of America; 4 Department of Statistics, University of Pretoria, Pretoria, South Africa; University Of Central Greece, GREECE

## Abstract

Meta-analysis of longitudinal studies combines effect sizes measured at pre-determined time points. The most common approach involves performing separate univariate meta-analyses at individual time points. This simplistic approach ignores dependence between longitudinal effect sizes, which might result in less precise parameter estimates. In this paper, we show how to conduct a meta-analysis of longitudinal effect sizes where we contrast different covariance structures for dependence between effect sizes, both within and between studies. We propose new combinations of covariance structures for the dependence between effect size and utilize a practical example involving meta-analysis of 17 trials comparing postoperative treatments for a type of cancer, where survival is measured at 6, 12, 18 and 24 months post randomization. Although the results from this particular data set show the benefit of accounting for within-study serial correlation between effect sizes, simulations are required to confirm these results.

## Introduction

In univariate meta-analysis, individual effect sizes such as odds ratios from two or more studies are combined into a single summary effect size. For instance, odds ratios from 33 randomized controlled trials evaluating the use of intravenous streptokinase for the treatment of myocardial infarction, consisting of a total of 36 974 participants, were pooled in a univariate meta-analysis [1]. Univariate meta-analysis has been applied in many fields of research such as pharmacology [2], psychology [3], education [4], and evidence-based medicine [5]. The methods for univariate meta-analysis are well-known ([6]–[15]) and it can be implemented in standard statistical software such as using STATA command **metan** [16], **metafor** package in R [17], and the **mixed** procedure in SAS [18]. There are also common routine computer packages that can perform univariate meta-analysis such as MetaWin [19], WEasyMA [20], Review Manager [21], MIX [22], Comprehensive Meta-analysis [23], and OpenMetaAnalyst ([24, 25]).

In the case where there are multiple correlated effect sizes per study, an analyst can either perform separate univariate meta-analysis for each effect size or perform multivariate meta-analysis where the multiple effect sizes are jointly synthesized. A typical example comes from hypertension trials where both systolic and diastolic blood pressure measurements are reported. Multivariate meta-analysis methods are well-known ([6], [26]–[29]) and can be implemented in standard statistical software ([30]–[34]). The problem with performing separate univariate meta-analysis is that it ignores correlation between the effect sizes and this can increase the standard error of point estimates [35]. Empirical and simulation-based comparisons of point estimates of binary outcomes between multivariate and univariate meta-analyses have shown that although generally the point estimates were comparable, the multivariate model with the discrete likelihood yielded smaller between study variance estimates and narrower prediction intervals for new outcomes [36], [37]. In the case of outcome reporting bias, where some studies in a meta-analysis partially report results, multivariate meta-analysis can reduce the impact of this bias when compared with univariate meta-analysis [38]. However, although multivariate meta-analysis can produce estimates with better statistical properties, it often requires making more assumptions which may therefore not result in the expected benefits of inference [39].

Perhaps a bigger challenge in meta-analysis is when the effect sizes are reported longitudinally. For example, consider the data analysed in [40] where studies reported the effect of deep-brain stimulation (DBS) in patients with Parkinson’s disease at 3, 6, 12 months or later after implantation of the stimulator. The challenge is to account for correlation between effect sizes, both within and between studies. This longitudinal meta-analysis can be viewed in the framework of multivariate meta-analysis [41]. Furthermore, the longitudinal meta-analysis can be set within the general linear mixed model framework [40] which offers more flexibility in specifying covariance structures between effect sizes, both within and between studies. In this paper, we adopted the approach in [40] but extended it to other combinations of covariance structures for the between and within study effect sizes. We used a practical application example of a meta-analysis of 17 randomized controlled trials comparing radiotherapy and chemotherapy versus radiotherapy alone for postoperative treatment of malignant gliomas, where survival is reported at 6, 12, 18, and 24 months post randomization [42]. The structure of the paper is as follows: section 2 consists of longitudinal meta-analysis models, section 3 contains estimation methods, section 4 covers the different covariance structures applied in this paper, section 5 describes the example used in this paper including results, and section 6 covers the discussion of the methodology and application results.

## Longitudinal meta-analysis model

We require a meta-analysis of *n* studies, denoted by *i* = 1, ⋯, *n*. Consider *T* longitudinal effect sizes per study denoted by *t* = 1, ⋯, *T*. So each study *i* yields *T* estimated effect sizes

***Y***_*i*_ = (*Y*_*i*1_, ⋯, *Y*_*it*_, ⋯, *Y*_*iT*_)′ such that

$$\begin{array}{c} Y_{it}=x_{it}^{\prime }\beta +z_{it}^{\prime }\delta_{i}+e_{it}. \end{array}$$ (1)

In this linear model, **x**_*it*_ is a *p* × 1 design vector of *p* fixed effects with corresponding regression coefficients contained in the *p* × 1 vector, ***β***. Likewise ***z***_*it*_ is a *q* × 1 design vector of *q*(≤*p*) random effects which are set in the *q* × 1 vector, ***δ***_*i*_. The last term of the model, *e*_*it*_, is the residual term associated with *Y*_*it*_.

Extending Eq (1) above gives the model for ***Y***_*i*_, that is,

$$\begin{array}{c} Y_{i}=X_{i}\beta +Z_{i}\delta_{i}+e_{i}, \end{array}$$ (2)

which is a general linear mixed model [43]. We assume, without loss of generality, a no-intercept model where ***X***_*i*_ is a *T* × *p* design matrix of *p* fixed effects, ***β*** is a *p* × 1 vector of fixed effect regression coefficients to be estimated, ***Z***_*i*_(⊆ ***X***_*i*_) is a *T* × *q* design matrix of *q* random effects ***δ***_*i*_ = (*δ*_*i*1_, ⋯, *δ*_*ij*_, ⋯, *δ*_*iq*_)′, and ***e***_*i*_ = (*e*_*i*1_, ⋯, *e*_*it*_, ⋯, *e*_*iT*_)′ is a vector of residuals. Effect sizes from different studies are assumed to be independent, that is, cov(*e*_*it*_, *e*_*mt*′_) = 0 when *i* ≠ *m* for time points *t*, *t*′ = 1, ⋯, *T*. We also assume that residuals and random effects are independent, cov(***e***_*i*_, ***δ***_*i*_) = 0.

Here we assume, without loss of generality, that the joint distribution of random effects is **0**-centered ***δ***_*i*_ ∼ *MVN*(**0**, **Σ**) (Multivariate Normal Distribution) where **Σ** is a *q* × *q* symmetric positive-definite variance-covariance matrix consisting of diagonal elements $\text{var}\left(\delta_{ij}\right)=\tau_{j}^{2}\left(j=1,...,q\right)$ and non-diagonal elements *ρ*_*jj*′_ *τ*_*j*_ *τ*_*j*′_ with *ρ*_*jj*′_ representing the correlation between random effects *δ*_*ij*_ and *δ*_*ij*′_. We also assume that the joint distribution of residuals is **0**-centered ***e***_*i*_ ∼ *MVN*(**0**, ***S***_*i*_) with *T* × *T* symmetric positive-definite variance-covariance matrix of ***S***_*i*_ consisting of diagonal elements $\text{var}\left(e_{it}\right)=\sigma_{it}^{2}$ and non-diagonal elements *ρ*_*itt*′_ *σ*_*it*_ *σ*_*it*′_, where *ρ*_*itt*′_ is the within-study serial correlation of effect sizes between time points *t* and *t*′. Therefore marginally ***Y***_*i*_ ∼ *MVN*(***X***_*i*_ ***β***, ***V***_*i*_) where $V_{i}=Z_{i}\Sigma Z_{i}^{\prime }+S_{i}$ is a symmetric positive-definite variance-covariance matrix. The within-study and between-study correlations between effect sizes are determined by the covariance structures imposed on ***S***_*i*_ and **Σ** respectively.

The goal of meta-analysis is to estimate the parameters in the vector ***β***. We also estimate variances $\left(\tau_{j}^{2}\right)$ and correlations (*ρ*_*jj*′_) between random effects, which are entries of **Σ**. For the purpose of ensuring identifiability, we regard the entries of ***S***_*i*_ as fixed and known constants although they are estimated in practice.

## Estimation of parameters

### Maximum Likelihood Estimation

Let ***α*** denote the vector of all variance and covariance parameters found in $V_{i}\left(\alpha \right)=Z_{i}\Sigma Z_{i}^{\prime }+S_{i}$ and ***θ*** = (***β***′,***α***′)′ be the *s*–dimensional vector of all parameters in the marginal model for ***Y***_*i*_. The marginal likelihood function is given by

$$\begin{array}{ccc} L_{ML}\left(\theta \right) & = & \prod_{i=1}^{n}{\{\left(2\pi \right)}^{-T/2}{\left|V_{i}\left(\alpha \right)\right|}^{-1/2}\\ & & \times \;\exp(-\frac{1}{2}{\left(Y_{i}-X_{i}\beta \right)}^{\prime }V_{i}^{-1}\left(\alpha \right)\left(Y_{i}-X_{i}\beta \right))\} \end{array}$$ (3)

The marginal log-likelihood function ℓ(***θ***) is then given by

$$\begin{array}{ccc} \log L_{ML}\left(\theta \right) & = & -\left(nT/2\right)\log\left(2\pi \right)-\left(n/2\right)\log(|V_{i}\left(\alpha \right)|)\\ & & -\;\left(1/2\right)\sum_{i=1}^{n}{\left(Y_{i}-X_{i}\beta \right)}^{\prime }V_{i}{\left(\alpha \right)}^{-1}\left(Y_{i}-X_{i}\beta \right) \end{array}$$ (4)

Assuming ***α*** to be known, the maximum likelihood estimator (MLE) of ***β***, obtained from maximizing Eq (4), conditional on ***α*** is then given by ([43], [44])

$$\begin{array}{c} \hat{\beta }\left(\alpha \right)={\left(\sum_{i=1}^{n}X_{i}^{\prime }W_{i}X_{i}\right)}^{-1}\sum_{i=1}^{n}X_{i}^{\prime }W_{i}Y_{i}, \end{array}$$ (5)

where $W_{i}=V_{i}^{-1}\left(\alpha \right)$.

In the case where ***α*** is not known, but an estimate $\hat{\alpha }$ is given, then ***β*** is estimated by Eq (5) with ***W***_*i*_ replaced by ${\hat{W}}_{i}=V_{i}^{-1}\left(\hat{\alpha }\right)$. The MLE of ***α*** is obtained by maximizing Eq (4) with respect to ***α***, after ***β*** is replaced by Eq (5).

### Restricted Maximum Likelihood Estimation

The Restricted Maximum Likelihood Estimators (REML) of ***α*** and ***β*** can be found by maximizing the REML likelihood function [44]

$$\begin{array}{c} L_{REML}\left(\theta \right)={\left|\sum_{i=1}^{n}X_{i}^{\prime }W_{i}\left(\alpha \right)X_{i}\right|}^{-\frac{1}{2}}L_{ML}\left(\theta \right) \end{array}$$ (6)

with respect to all parameters (***α*** and ***β***) simultaneously.

## Modeling covariance structures

For brevity and without loss of generality, we assume *T* = 4 time points for each study. We also assume, for parsimonious reasons and without loss of generality, that ***X***_*i*_ consists of only time indicators such that ***X***_*i*_ = ***I***_4_ (an identity matrix of order 4), where we ignore intercept terms. We consider six models with different covariance structures for Eq (2).

### Model 1 - Independent random time effects model

In this model, effect sizes at different time points do not depend on each other. It is therefore equivalent to performing univariate random effects meta-analysis at each time point separately. Mathematically this model allows independent random intercept effects at each time point *t* per study *i*, *δ*_*it*_, such that

$$\begin{array}{c} Y_{it}=\beta_{t}+\delta_{it}+e_{it},t=1,\cdots ,4, \end{array}$$ (7)

where we assume $\delta_{it}∼N\left(0,\tau_{t}^{2}\right)$ and $e_{it}∼N\left(0,\sigma_{it}^{2}\right)$ to be independent. We therefore set ***Z***_*i*_ = ***X***_*i*_ = ***I***_4_ so that Eq (2) becomes

$$\begin{array}{c} Y_{i}=\beta +\delta_{i}+e_{i}, \end{array}$$ (8)

and

$$\begin{array}{c} V\left(Y_{i}\right)=\Sigma +S_{i}=\text{diag}\left(\tau_{1}^{2}+\sigma_{i1}^{2},\tau_{2}^{2}+\sigma_{i2}^{2},\tau_{3}^{2}+\sigma_{i3}^{2},\tau_{4}^{2}+\sigma_{i4}^{2}\right). \end{array}$$ (9)

However, this model ignores within-study serial correlation between longitudinal effect sizes which exists because it is the same individuals who are measured repeatedly at these time points.

### Model 2 - Random study effects model

This model accounts for dependence between effect sizes by assigning a random intercept effect that is common to all longitudinal effect sizes from a given study while assuming zero within-study serial correlations between longitudinal effect sizes, that is, $S_{i}=\text{diag}\left(\sigma_{i1}^{2},..,\sigma_{i4}^{2}\right)$. Therefore ***Z*** is a 4 × 1 vector of ones so that ***δ***_*i*_ = *δ*_*i*_ is a scalar and the model is now given by

$$\begin{array}{c} Y_{it}=\beta_{t}+\delta_{i}+e_{it},t=1,\cdots ,4, \end{array}$$ (10)

where we assume *δ*_*i*_ ∼ *N*(0, *τ*^2^) with *τ*^2^ representing the between-study variability or heterogeneity. The variance-covariance matrix is now given by $V\left(Y_{i}\right)=Z_{i}\Sigma Z_{i}^{\prime }+S_{i}$, a 4 × 4 matrix consisting of diagonal elements set to $\tau^{2}+\sigma_{it}^{2}$ and off-diagonal elements all equal to *τ*^2^, where **Σ** = *V*(*δ*_*i*_) = *τ*^2^. Since all the off-diagonal elements are equal to *τ*^2^, we can deduce that $\text{corr}\left(Y_{it},Y_{it^{\prime }}\right)=\tau^{2}/\sqrt{\left(\tau^{2}+\sigma_{it}^{2}\right)\left(\tau^{2}+\sigma_{it^{\prime }}^{2}\right)}$ between two time points (*t*, *t*′). Therefore, by including a random study effect, we automatically induce a correlation between any two effect sizes within a study. These correlations are assumed to be the same for each set of time points, regardless of the time lag between the time points. This covariance structure is also known as compound symmetry.

However, this model allows only one random effect for all the longitudinal effect sizes from each study and therefore ignores the serial correlation between effect sizes for instance, effect sizes closer together tend to be more strongly correlated than those measured far apart due to factors such as loss-to-follow-up.

### Model 3 - Correlated random time effects model

This is an extension of the independent random time effects model where the dependence between effect sizes is accounted for through the dependence between random time effects. This model imposes heteroscedastic AR(1) covariance structure for the random time effects while assuming zero within-study serial correlations between longitudinal effect sizes, that is, $S_{i}=\text{diag}\left(\sigma_{i1}^{2},..,\sigma_{i4}^{2}\right)$. As a result, the variance-covariance matrix is now given by *V*(***Y***_*i*_) = **Σ** + ***S***_*i*_, with diagonal elements ($\tau_{1}^{2}+\sigma_{i1}^{2},..,\tau_{4}^{2}+\sigma_{i4}^{2}$) and off-diagonal elements ($\rho_{\tau }^{|t-t^{\prime }|}\tau_{t}\tau_{t^{\prime }}$) for time points *t* and *t*′, where *ρ*_*τ*_ is the correlation between any two adjacent random time effects. Therefore the dependence between effect sizes become stronger as the lag between them gets smaller. This is plausible in longitudinal studies where loss-to-follow up increases with time such that effect sizes measured far apart have less dependence than those closer to one another.

However, this model assumes independent within-study residuals which is not suitable for longitudinal effect sizes. A structure that takes account of the autocorrelation between the effect sizes within a study is more suitable.

### Model 4 - Correlated within-study effect sizes model

This is an extension of the independent random time effects model where the dependence between effect sizes is accounted for through the dependence in effect sizes within the same study. This model imposes heteroscedastic AR(1) covariance structure for the within-study longitudinal effect sizes while assuming independent random time effects, that is, $\Sigma =\text{diag}(\tau_{1}^{2},..,\tau_{4}^{2})$. As a result, the variance-covariance matrix is now given by *V*(***Y***_*i*_) = **Σ** + ***S***_*i*_, with diagonal elements ($\tau_{1}^{2}+\sigma_{i1}^{2},..,\tau_{4}^{2}+\sigma_{i4}^{2}$) and off-diagonal elements ($\rho_{s}^{|t-t^{\prime }|}\sigma_{it}\sigma_{it^{\prime }}$) for time points *t* and *t*′, where *ρ*_*s*_ is the correlation between any two adjacent within-study effect sizes.

This model, which imposes correlated within-study effect sizes while assuming independent random time effect, was not applied in either [40] or [41]. The purpose of including this model is to assess which covariance structure results in a more improved model between the within-study covariance matrix (***S***_*i*_) and between-study covariance (**Σ**).

### Model 5 - Correlated within-study effect sizes and correlated random time effects

This is an extension of the independent random time effects model where the dependence between effect sizes is accounted for through the dependence in both effect sizes within the same study and random time effects. It is a combination of the above two models, where the heteroscedastic AR(1) covariance structures are imposed on both ***S***_*i*_ and **Σ**. The variance-covariance matrix is now given by *V*(***Y***_*i*_) = **Σ** + ***S***_*i*_, with diagonal elements ($\tau_{1}^{2}+\sigma_{i1}^{2},..,\tau_{4}^{2}+\sigma_{i4}^{2}$) and off-diagonal elements ($\rho_{\tau }^{|t-t^{\prime }|}\tau_{t}\tau_{t^{\prime }}+\rho_{s}^{|t-t^{\prime }|}\sigma_{it}\sigma_{it^{\prime }}$) for time points *t* and *t*′.

This model accounts for any dependence between effect sizes, both within and between studies. However, this model requires estimation of one more parameter compared to each of the above two models.

### Model 6 - Correlated random time effects (unstructured) and correlated within-study effect sizes

This is an extension of the independent random time effects model where the dependence between effect sizes is accounted for through the dependence in both effect sizes within the same study and random time effects. We assume an unstructured covariance structure for the random time effects and a heteroscedastic AR(1) covariance structure for the within-study longitudinal effect sizes. The variance-covariance matrix is now given by *V*(***Y***_*i*_) = **Σ** + ***S***_*i*_, with diagonal elements ($\tau_{1}^{2}+\sigma_{i1}^{2},..,\tau_{4}^{2}+\sigma_{i4}^{2}$) and off-diagonal elements ($\rho_{tt^{\prime }}\tau_{t}\tau_{t^{\prime }}+\rho_{s}^{|t-t^{\prime }|}\sigma_{it}\sigma_{it^{\prime }}$) for time points *t* and *t*′.

This combination of covariance structures was also not applied by [40] and [41]. The unstructured covariance matrix is quite a superior covariance structure although its requirement for a higher number of parameters may compromise model parsimony and convergence in some cases.

We did not include models with heteroscedastic compound symmetry (CSH) and autoregressive of order 1 (AR(1)) because we obtained similar results to the models above.

## Example

We use the example given in [42], also used by [41], of a meta-analysis of 17 randomized controlled trials comparing post-operative radiation therapy plus chemotherapy (Experimental group (E)) with radiation therapy alone (Control group (C)) in patients with malignant gliomas. The outcome of interest is the number of patients surviving at 6, 12, 18, and 24 months. The data, as given and described in [41], is reproduced in Table 1. We use this example to illustrate the efficiency of the longitudinal meta-analysis models described above. However, since the meta-analysis data is not up-to-date, we will not focus on the clinical significance of these treatments for this condition.

**Table 1:** Number of survivors at 6, 12, 18 and 24 months following post-operative treatment with either radiotherapy plus chemotherapy (E) or radiotherapy alone (C) in patients with malignant gliomas from 17 studies [42].

Study	Sample size, E(C)	Number of survivors, E(C)			
		6 months	12 months	18 months	24 months
1	19 (22)	16 (20)	11 (12)	4 (8)	4 (3)
2	34 (35)	22 (22)	18 (12)	15 (8)	15 (6)
3	72 (68)	44 (40)	21 (15)	10 (3)	3 (0)
4	22 (20)	19 (12)	14 (5)	5 (4)	2 (3)
5	70 (32)	62 (27)	42 (13)	26 (6)	15 (5)
6	183 (94)	130 (65)	80 (33)	47 (14)	30 (11)
7	26 (50)	24 (30)	13 (18)	5 (10)	3 (9)
8	61 (55)	51 (44)	37 (30)	19 (19)	11 (15)
9	36 (25)	30 (17)	23 (12)	13 (4)	10 (4)
10	45 (35)	43 (35)	19 (14)	8 (4)	6 (0)
11	246 (208)	169 (139)	106 (76)	67 (42)	51 (35)
12	386 (141)	279 (97)	170 (46)	97 (21)	73 (8)
13	59 (32)	56 (30)	34 (17)	21 (9)	20 (7)
14	45 (15)	42 (10)	18 (3)	9 (1)	9 (1)
15	14 (18)	14 (18)	13 (14)	12 (13)	9 (12)
16	26 (19)	21 (15)	12 (10)	6 (4)	5 (1)
17	74 (75)	–	42 (40)	–	23 (30)

There were missing data for study 17 at months 6 and 18. There were no survivors in the control group at month 24 for studies 3 and 10.

### Estimation and implementation of the models

The parameters for the general linear mixed model were estimated using the restricted maximum likelihood (REML) estimation in R. The R code is given in Appendix A. However, for the estimation of within-study error correlation, a SAS program by [40] was used.

### Results for the separate univariate random effects meta-analysis

**Fig 1:**
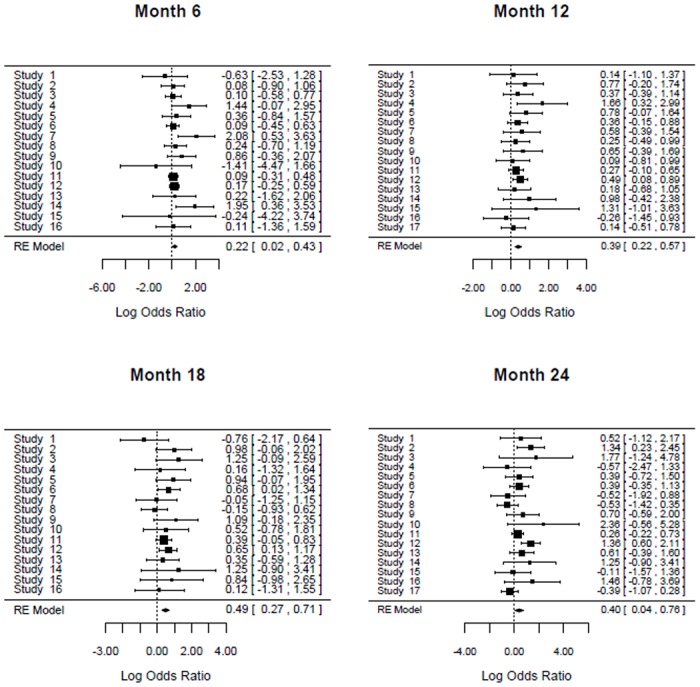
Forest Plots for Month 6, 12, 18 and 24.

We first ran separate univariate random effects meta-analyses for month 6, 12, 18 and 24. The results obtained are summarised in Table 2 and forest plots are given in Fig 1.

**Table 2:** Meta-analysis results from separate univariate random effects meta-analyses for the log odds ratio of surviving under the experimental (E) versus the control (C) treatments at month 6, 12, 18, and 24 [42].

	log OR (95%CI)	*τ*^2^	*χ*^2^ p-value	*I*^2^
Month 6	0.22 (0.02, 0.43)	0.00	0.348	0.0%
Month 12	0.39 (0.22, 0.57)	0.00	0.876	0.0%
Month 18	0.49 (0.27, 0.71)	0.00	0.661	0.0%
Month 24	0.40 (0.04, 0.76)	0.20	0.053	42.0%

The results in Table 2 clearly shows that the odds of survival were significantly higher in the experimental group compared with the control group. This was consistent across all longitudinal time points from month 6 to month 24. All the four log odds ratios at month 6, 12, 18 and 24 months were statistically significant because the 95% confidence intervals are all greater than 0. The least log odds ratio (0.22) was at month 6 which increased at month 12 (0.39) and at month 18 (0.49). This highest log odds ratio at month 18 slightly decreased to 0.40 at month 24. Heterogeneity was not statistically significant at all the time points ([12], [45], [46]).

### Results for the linear mixed model

The results of applying the general linear mixed model Eq (2) to the example data using models 1 to 6 are shown in Tables 3 and 4. We obtained exactly the same results for the independence model as the ones obtained from the separate meta-analyses in Table 2. This is because the independence model is equivalent to performing univariate meta-analysis at each time point separately. Inspection of the log odds ratio estimates from all the six models (Tables 3 and 4) show slight differences between the models. It is also clear that the pattern of the results were the same across all the six models; the log odds ratios at month 6 were the least, they increased at month 12 and increased again to reach maximum at month 18 after which they decreased slightly at month 24. All the log odds ratio estimates at month 12 and 18 showed that the odds of surviving were significantly higher for the experimental treatment compared to the control treatment since the 95% confidence intervals exceed the value of 0. This statistical significance was also shown for month 6 using models 1,3, and 6. Although the log odds ratios at month 6 for models 2, 4 and 5 are not statistically significant at 5% level of significance, the corresponding p-values were slightly above 0.05 (data not shown). At month 24, the odds of surviving under experimental versus control treatment were higher for all the models except models 5 and 6 where the p-values were also slightly above 0.05. In overall, the likelihood of survival is significantly better under the experimental treatment compared to the control treatment.

**Table 3:** Meta-analysis results for models 1 to 3 from the linear mixed model for the log odds ratio of surviving under experimental treatment compared to the control treatment using data for 17 trials [42].

	Model 1	Model 2	Model 3
Covariance structures Between random time effects (Σ)	Indep^*a*^	CS^*b*^	HAR(1)^*c*^
Within-study errors (*S*_*i*_)	Indep	Indep	Indep
Log odds ratio estimates			
Month 6	0.22 (0.02, 0.43)	0.22 (-0.01, 0.45)	0.22 (0.02, 0.43)
Month 12	0.39 (0.22, 0.57)	0.41 (0.21, 0.61)	0.39 (0.21, 0.57)
Month 18	0.49 (0.27, 0.71)	0.50 (0.25, 0.74)	0.47 (0.21, 0.72)
Month 24	0.40 (0.04, 0.76)	0.38 (0.12, 0.65)	0.42 (0.05, 0.78)
Between study variance estimates			
Month 6	0.00	*τ*^2^ = 0.03	0.00
Month 12	0.00		0.01
Month 18	0.00		0.05
Month 24	0.20		0.23
Model Fit			
AIC^d^	121.3	119.6	120.5

^*a*^Indep = Independence

^*b*^CS = Compound Symmetry

^*c*^HAR(1) = Heteroscedastic autoregressive (1)

^d^AIC = Akaike Information Criterion

**Table 4:** Meta-analysis results for models 4 to 6 from the linear mixed model for the log odds ratio of surviving under experimental treatment compared to the control treatment using data for 17 trials [42].

	Model 4	Model 5	Model 6
Covariance structures Between random time effects (Σ)	Indep^*a*^	HAR(1)^*b*^	UN^*c*^
Within-study errors (*S*_*i*_)	HAR(1)	HAR(1)	HAR(1)
Log odds ratio estimates			
Month 6	0.18 (-0.02, 0.38)	0.18 (-0.02, 0.38)	0.21 (0.00, 0.42)
Month 12	0.35 (0.18, 0.52)	0.35 (0.17, 0.52)	0.35 (0.18, 0.53)
Month 18	0.41 (0.19, 0.62)	0.39 (0.15, 0.62)	0.38 (0.15, 0.62)
Month 24	0.37 (0.05, 0.69)	0.35 (-0.01, 0.72)	0.34 (-0.03, 0.71)
Between study variance estimates			
Month 6	0.00	0.00	0.01
Month 12	0.00	0.00	0.00
Month 18	0.00	0.03	0.03
Month 24	0.13	0.23	0.23
Model Fit			
AIC^d^	106.9	107.2	116.7

^*a*^Indep = Independence

^*b*^HAR(1) = Heteroscedastic autoregressive (1)

^*c*^UN = Unstructured

^d^AIC = Akaike Information Criterion

Results for the between-study variances from the linear mixed model ranged from 0.00 to 0.23 and were not statistically different from zero. Results for the estimates of within-study correlation are not shown in Tables 3 and 4 but we found values of 0.60 and 0.61 for models 4 and 5, respectively, using SAS code from [40].

The model fit as shown by the values of Akaike Information Criterion (AIC), where smaller values indicate better fit, show that models 4 and 5 had much better fit than the rest of the models. Models 2, 3 and 6 performed slightly better than the independence model and there were very slight differences in the model fit between these four models. Some points can be deduced from these results, at least for this particular data set: (1)accounting for correlation between effect sizes through either the random study effect model or the correlated random time effects model yield similar results to the independence model where separate meta-analyses are done at each time point; (2) results from models 4 and 5 clearly shows the benefit of accounting for within-study serial correlations between effect sizes and the fact that model 4 performed better than model 5 strengthens this finding; and (3) the confidence intervals for parameter estimates show that the best performing model 4 had the narrowest confidence intervals compared to the other five models at all the four time points.

## Discussion

This paper addresses the problem of estimating parameters for the meta-analysis of longitudinal studies. In the case where a summary measure such as an incidence rate is reported by each longitudinal study, a univariate meta-analysis of the incidence rates can be done and as an example, we carried out a meta-analysis of incidence rates of pregnancy among young women participating in vaginal microbicide trials for HIV prevention [47]. However, in cases where an effect size is reported at each one of multiple pre-determined time points, a multivariate meta-analysis [41] or the general linear mixed model [40] can be used to estimate overall effect sizes at each time point, while taking account of any correlation between effect sizes, both within and between studies. The general linear mixed model has an added advantage of more flexibility in specifying covariance structures for within- and between-studies. In this paper, we applied the general linear mixed model to an example from [42] of a meta-analysis of odds ratios from 17 trials for survival under experimental compared to control treatment. The simple approach of not accounting for the correlation, that is, the independence model where separate meta-analyses were done at each of the time points was contrasted against models where correlation was accounted for in different alternatives; including random study effects, correlated random time effects and/or correlated within-study errors, or unstructured covariance structures. This paper has proposed new combinations of covariance structures (models 4 and 6) that were not applied in either [41] or [40]. The results of all the six models applied in this paper consistently showed that the odds of survival under the experimental treatment were significantly higher compared to the control treatment across all the longitudinal time points from month 6 to month 24 after treatment. Our results are consistent with the results from [41], in which the authors applied the multivariate meta-analysis model to the same data [42]. The model that performed best was the one that accounted for within-study serial correlation between effect sizes using the heteroscedastic autoregressive structure. Accounting for this correlation using the compound symmetry showed very little benefit compared to the independence model. In this particular longitudinal data set, the autoregressive covariance structure yielded more precise estimates compared to the compound covariance structure.

Simulations to confirm whether our findings of the benefit of taking account of within study correlations using the autoregressive structure are needed since our results only apply to our particular data set and cannot be generalized to other data sets. In this study, we have also not explored whether our multivariate models would improve our point estimates in the presence of missing data since our example data set had very minimal missing data. In the literature, some studies have shown that, in the presence of large amount of missing data, the ‘borrowing of strength’ from other studies in a multivariate meta-analysis can give more precise estimates compared to the univariate meta-analysis [39], [48], [49]. This has also been shown in a particular case of outcome reporting bias, where the impact of this bias on the precision of point estimates was reduced in the multivariate meta-analysis compared to univariate meta-analysis [38]. This could be explained by the fact that multivariate meta-analysis takes account of the correlation between the outcomes and thereby adds information on the missing outcomes [37]. Although this evidence may imply that a multivariate meta-analysis can be used to jointly meta-analyze outcomes when there are missing values without further need for imputations, simulations are needed to show it.

This paper has potential to be extended in some respects. Our modeling approach was to estimate point estimates at each fixed time point. The alternative approach is to treat time as a continuous covariate and explore both linear and non-linear models as shown in Ahn and French (2010) [50]. This may improve the estimation and is a subject of further study. The models and analyses in this paper could be extended to where effect sizes are not necessarily assumed to be normal. The methodology in this paper can also be extended where at each time point, we have multiple effects sizes of different types. Though this creates complexity in the modelling structures, such extensions are well suited in the prevailing longitudinal studies where a number of outcomes are measured at multiple time points. We are currently working on these methodological extensions.

## Supporting Information

S1 Fig R-code for meta-analysis. (TIFF)
