# Supplementary figures and images for: Meta-Analysis of Effect Sizes Reported at Multiple Time Points Using General Linear Mixed Model
Source: PLoS One. 2016 Oct 31;11(10):e0164898. doi: 10.1371/journal.pone.0164898 (PMC5087886; doi:10.1371/journal.pone.0164898)

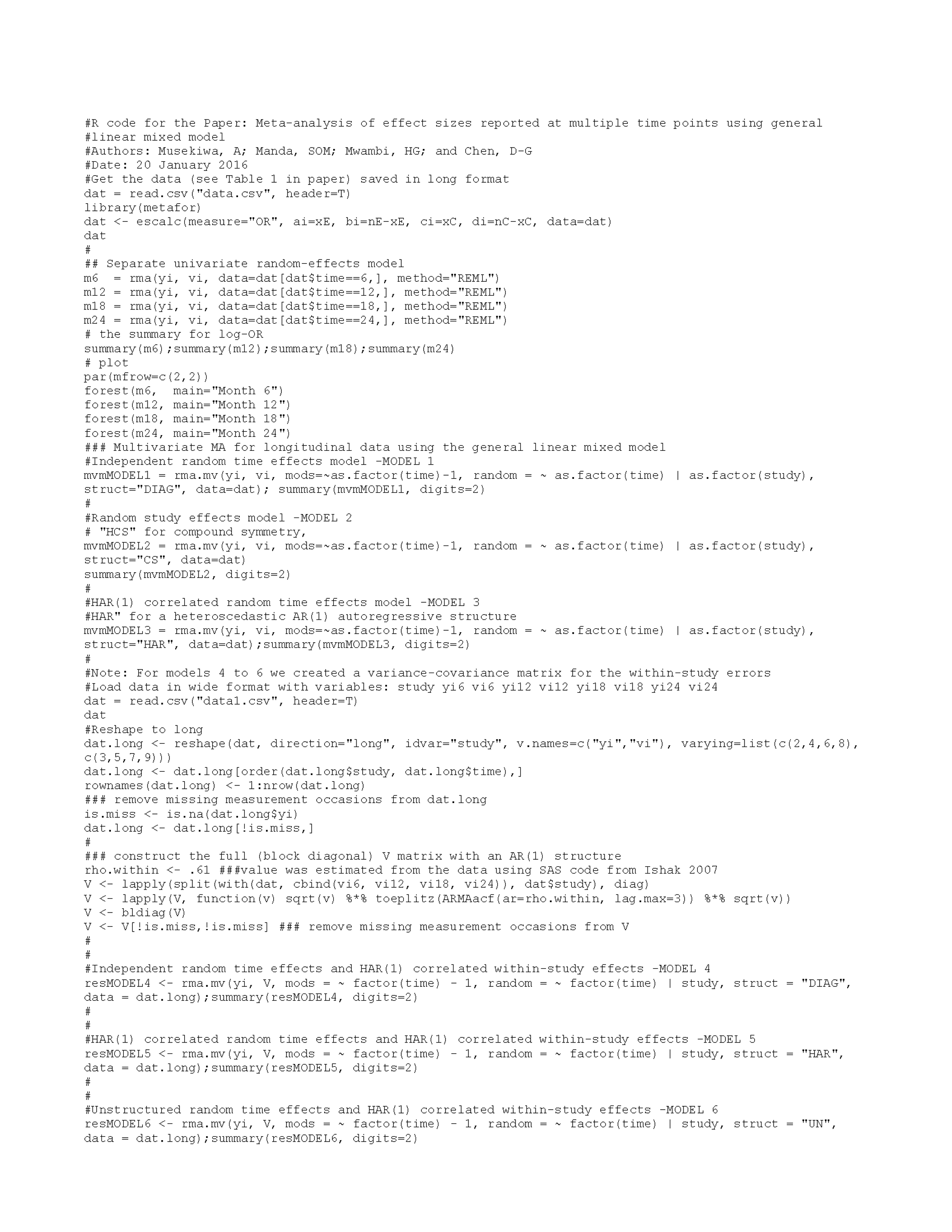

Supplement: S1 Fig — (TIFF) [file pone.0164898.s001.tiff]
